# Circulating plasma-derived extracellular vesicles expressing bone and kidney markers are associated with neurocognitive impairment in people living with HIV

**DOI:** 10.3389/fneur.2024.1383227

**Published:** 2024-04-23

**Authors:** Erika G. Marques de Menezes, Scott A. Bowler, Cecilia M. Shikuma, Lishomwa C. Ndhlovu, Philip J. Norris

**Affiliations:** ^1^Vitalant Research Institute, San Francisco, CA, United States; ^2^Department of Laboratory Medicine, University of California, San Francisco, San Francisco, CA, United States; ^3^Division of Infectious Diseases, Department of Medicine, Weill Cornell Medicine, New York, NY, United States; ^4^Hawaii Center for AIDS, John A. Burns School of Medicine, University of Hawaii, Honolulu, HI, United States; ^5^Department of Tropical Medicine, John A. Burns School of Medicine, University of Hawaii, Honolulu, HI, United States; ^6^Department of Medicine, University of California, San Francisco, San Francisco, CA, United States

**Keywords:** extracellular vesicles, bone and kidney cells, cognitive impairment, human immunodeficiency virus, antiretroviral therapy

## Abstract

**Background:**

Although effective antiretroviral therapy (ART) has improved the life expectancy of people with HIV (PWH), the prevalence of milder forms of HIV-associated neurocognitive disorders (HAND) persist, and it is associated with systemic and neuro-inflammatory processes that could impact other organ systems. However, the complex signaling mechanisms between the bone-kidney systems and the brain in HAND remain unknown. Extracellular vesicles (EVs) play a potential role in inter-organ communication and are involved in regulating cell activity in distant tissues. In this study, we examined whether levels of EVs from bone-and kidney-related cells associate with cognitive dysfunction and explored the relationship between kidney-bone EV axis in PWH experiencing cognitive deficits.

**Methods:**

EV subtypes were characterized in plasma from 61 PWH with either cognitive impairment (CI, *n* = 53) or normal cognition (NC, *n* = 8) based on the American Academy of Neurology criteria for HIV-associated dementia (HAD, *n* = 11), minor cognitive motor disorder (MCMD, *n* = 25) or asymptomatic neurocognitive impairment (ANI, *n* = 17) by spectral flow cytometry. EVs were profiled with markers reflecting bone and kidney cell origin. A support vector machine learning-based model was employed for analyses of EV phenotypes to predict the cognitive dysfunction.

**Results:**

Plasma-EVs expressing osteocalcin, sclerostin, and nephrin were significantly higher in the cognitive impairment group compared to the normal cognition group. EVs bearing kidney cell markers correlated significantly with bone-derived EVs. A machine learning-based model, comprised of osteocalcin+, nephrin+, and CD24+ EVs predicted cognitive impairment in PWH on ART.

**Conclusion:**

Our study reveals that neurocognitive impairment in PWH is associated with increased levels of plasma EVs enriched with the bone markers osteocalcin and sclerostin and the kidney marker nephrin, suggesting that these EV subtypes may be novel candidate biomarkers for disease-spanning neurocognitive dysfunction. Moreover, the relationship between bone-derived EVs with kidney-derived EVs may suggest their role in mediating inter-organ crosstalk in the pathogenesis of HIV-associated cognitive impairment.

## Introduction

Despite long term virological suppression by effective antiretroviral therapy (ART), milder forms of cognitive impairment, including asymptomatic neurocognitive impairment (ANI) and minor cognitive motor disorder (MCMD) continue to affect 10–30% of people with HIV (PWH) (1, 2). The pathophysiological mechanisms are still unclear but probably are associated with persistent immune activation and inflammation, blood–brain barrier dysfunction, metabolic changes, drugs of abuse, secondary effects of aging, and virus-induced neurotoxicity (3, 4). Furthermore, the pathogenesis of HIV-associated neurocognitive impairment in the era of ART appears to be multifactorial with contributions from various age-related comorbidities, including osteoporosis and renal disease (5–7). To our knowledge, only a few studies have reported the association between bone or kidney markers and cognitive impairment in PWH (8–10), emphasizing the need for further investigation to explore potential links between the bone-kidney axis and cognitive function in the context of HIV infection. However, understanding disease mechanisms that drive cognitive impairment in PWH and identifying promising molecular targets and putative biomarkers has been challenging.

In recent years increasing attention has been given to the potential role of extracellular vesicles (EVs) in driving or modulating immune responses and cell-pathogen interactions (11). EVs have the ability to cross biological barriers to transfer functional proteins, viral proteins, microRNA (miRNAs), or other EV cargo to neighboring cells and mediate intercellular and inter-organ crosstalk between the brain and other organs (12). We recently showed that EVs expressing monocyte activation and neuronal markers associated with HIV-related neurocognitive impairment. We further showed that circulating platelet EV levels were linked to monocyte activation indicating a potential interaction in the pathological processes involved in HIV-associated neurological disorders (13). Interestingly, a study performed by Jiang et al. (14) showed that inhibition of young osteocyte-derived EVs contributed to accelerated cognitive impairment in an Alzheimer’s disease mouse model. Another study reported that urinary EVs expressing calbindin, a protein expressed in the kidney and brain, were associated with Parkinson’s disease individuals (15). In addition, it was found that miR-483-5p enriched EVs correlated with the bone loss in Alzheimer’s disease individuals, suggesting that EV miR-483-5p may serve as a mediator of neuronal control of bone remodeling (16). Given these findings, together with evidence of the communication between the brain and other organs, it would be advantageous to focus on EVs carrying the surface proteins from specific parent cell types that may be engaged in the pathophysiology to narrow down off-target effects and provide valuable insights into the underlying disease mechanisms. Furthermore, EVs not only influence disease through cellular crosstalk but can also carry disease-specific signatures and mimic the status of their cell of origin. However, to our knowledge, there are no published human studies that have explored the role of EVs as key mediators in the crosstalk between the bone/kidney axis in PWH with cognitive impairment. This study assessed the interactions between the brain and bone/kidney systems in the context of HIV infection and cognitive impairment by determining the changes in circulating levels of EVs expressing bone and kidney related markers. Furthermore, we assessed the relationship of organ-derived EVs with each other based on surface markers from their cell of origin.

## Methods

### Study participants and sample collection

This study utilized stored plasma samples from the Hawaii Aging with HIV Cohort (HAHC) study, a longitudinal cohort designed to investigate the impact of age and HIV on cognitive function (17). At enrollment, demographic information, clinical laboratory data, and medical history were obtained. The cohort details have been published previously (18). This study was approved by the Institutional Review Board of the University of Hawaii Committee on Human Subjects, and all participants signed a written informed consent.

Sixty-one plasma samples from PWH were selected for EV analysis based on sample availability. Participants were classified with either normal cognition (*n* = 8) or cognitive impairment based on the American Academy of Neurology (AAN) criteria for HIV-associated dementia (HAD, *n* = 11), minor cognitive motor disorder (MCMD, *n* = 25) or asymptomatic neurocognitive impairment (ANI, *n* = 17) (19). Blood was collected in an EDTA tube and centrifuged at 2000 g for 10 min at 4°C, and the obtained plasma was stored at −80°C until further analysis.

### Neurocognitive evaluation

The 80-min neuropsychological battery used within the HAHC has previously been described (18). Briefly, this battery was designed to assess multiple cognitive domains affected by HIV-infection including Choice and Sequential Reaction Time (CalCap), Rey Auditory Verbal Learning Test (RAVLT), Rey-Osterreith Complex Figure (RCF) Copy and Recall, Trail Making Tests A and B, WAIS-R Digit Symbol, Grooved Pegboard, Timed Gait, Odd Man Out, FAS, Animal Naming, Boston Naming Test (BNT), and WAIS-R Digit Span (Forward and Backward). Scores were converted to normative-adjusted z-scores from HIV seronegative subjects with similar risk profiles as the HAHC. A sum score (NPZ8) was constructed from the following tests: RAVLT trial 5, delayed recall and delayed recognition, RCF immediate recall and copy, Odd Man Out, FAS, Digit Symbol, CalCap choice and sequential reaction times, and Grooved Pegboard dominant and non-dominant hands. NPZ8 was used as a comparative, secondary assessment of neurocognitive impairment, but was not a determinate in NC/CI classification. All neuropsychological testing was performed by an examiner trained and supervised by a clinical neuropsychologist.

### EV characterization by spectral flow cytometry

**Figure 1:**
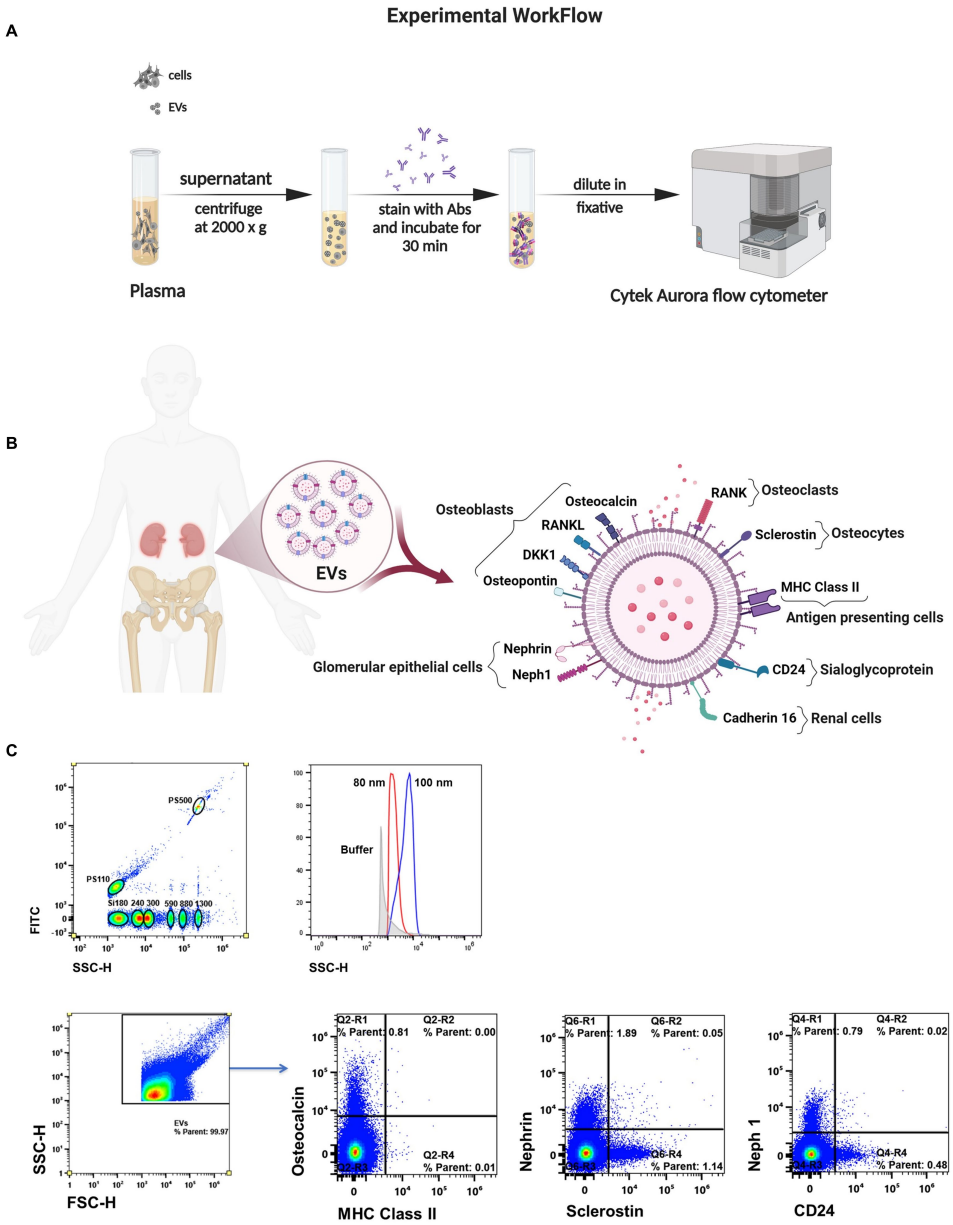
Measuring single EVs directly in biofluid. **(A)** Schematic of the method for isolation and spectral characterization of EVs. **(B)** Illustration of surface markers on EVs linked to bone cells and kidney cells. **(C)** Sensitivity to discriminate beads ≥80 nm in diameter by violet laser scatter (SSC). Representative flow plots of EV subtypes according their cell of origin.

To determine EV quantity and putative cell source markers, plasma samples were thawed and promptly processed to minimize the impact of freeze–thaw cycles. EVs were isolated by centrifugation at 2000 g for 15 min at 4°C and designated as large EVs in accordance with an ISEV position paper (20). As is known, EV is an umbrella term that encompasses smaller EVs (which would include exosome markers) and larger EVs, which can be generated from the cell surface, and there are no universal molecular markers for EV subtypes generated from the cell surface (20, 21). However, the selection of putative surface markers was guided by the source cell origin. Large EVs were stained using pre-titrated volumes of fluorochrome-conjugated monoclonal antibodies in two separate panels, purchased from Santa Cruz Biotechnology unless otherwise noted: osteoblasts osteocalcin-PE, osteopontin-Alexa Fluor 790, DKK1 (dickkopf-1)-Alexa fluor 594, and RANKL (receptor activator of NF-kB ligand)-Alexa Fluor 647, osteocytes sclerostin-Alexa Fluor 488, osteoclasts RANK (receptor activator of NF-kB)-Alexa Fluor 488, glomerular epithelial cells nephrin-Alexa Fluor 647, and neph1-Alexa Fluor 790, renal cells cadherin16-PE, sialoglycoprotein CD24-BV421 (BioLegend), and antigen-presenting cells MHC Class II (major histocompatibility complex class II)-BV510 (BioLegend) (Figures 1A,B). Fluorescence minus one controls were used to determine background signal. Antibodies were filtered using a using a pore size of 0.22 μm centrifugal filter (Millipore) to remove aggregates. One to 2 μL were added to 10 μL of EVs and incubated at 4°C for 30 min. EVs were diluted in 0.22 μm-filtered PBS containing 2.8% formaldehyde (BD stabilizing fixative) to appropriate dilutions to avoid coincident detection, as previously described (22).

Considering the methodological guidelines for EV studies (20, 21), large EVs were characterized based on their cellular origin using a high-sensitivity spectral flow cytometer with five lasers (UV, violet, blue, yellow/green, and red; Cytek Biosciences).The MIFlowCyt checklist was included as Supplemental material. Quality control was performed using SpectroFlo QC Beads according to the manufacture’s instructions (Cytek Biosciences). A clean flow cell procedure was conducted prior to sample analysis to minimize EV carryover. A 0.22 μm-filtered PBS control was recorded to estimate the background noise. Unstained samples were used under the same conditions as the stained EV samples to ensure accurate unmixing and autofluorescence quantitation by flow cytometry. Side scatter was measured using the 405 nm violet laser at a threshold of 1,000 arbitrary units. Samples were acquired for 60 s at a low flow rate (~15 μL/min) and a double SIT flush was performed over the wash station after each acquisition. The reference bead mix (Apogee Flow Systems) composed of a mixture of 110 nm and 500 nm polystyrene (PS) beads, and 180, 240, 300, 590, 880, and 1,300 silica beads were used to evaluate the fluorescence performance. EV gates were established using PS beads ranging from 80 nm to 1,300 nm (Apogee and NIST-traceable PS beads). Representative flow cytometry plots showing the expression of surface markers are shown in Figure 1C. EV counts/μL were calculated using the flow rate of the cytometer. Analysis was performed using SpectroFlo software (Cytek Biosciences).

### Statistical analysis

Participant demographic and clinical characteristics are represented as frequencies or medians with interquartile range (IQR) for categorical and continuous variables, respectively. EV measures were log_10_ transformed and Shapiro–Wilk test was used to assess normality for all variables prior to statistical analysis. Comparisons between CI sub-groups (ANI/MCMD/HAD) were performed by Kruskal-Wallis tests, while chi-squared test was used to examine categorical differences. Mann–Whitney U test was used to examine unpaired differences of continuous measures. XGBoost modeling was used to assess the probability of cognitive status. XGBoost model was trained on a random sampling of 70% of the dataset, while the remaining 30% were reserved as a validation group. Receiver operating characteristic (ROC) plots were used to graphically represent model performance. Given the imbalance between classes (NC vs. CI) and the sensitivity of ROC area under the curve (AUC) has to class imbalance, accuracy score (sklearn v.1.2.0) was used to evaluate model performance. Spearman correlation coefficients were used to assess pairwise relationships between variables. The Benjamini-Hochberg procedure was used to adjust for multiple comparisons. We used a *p*-value of ≤0.05 for statistical significance and employed an adjusted *p*-value <0.1 as the significance threshold for multiple comparisons, a choice considered acceptable given the aims of the study, as previously described (13).

## Results

### Demographics and clinical characteristics

Study participants’ demographic and clinical characteristics are shown in Table 1. A significant difference was found among the ANI, MCMD, and HAD groups for sex, CD4+ T cell count, and nadir CD4+ T cell count (*p* ≤ 0.034), while no difference were found for these variables between the NC and CI groups. There were no significant differences in age, viral load, or the duration of ART therapy between the NC, ANI, MCMD, and HAD groups. NPZ8 score was significantly higher in the NC group (*p* < 0.001).

**Table 1:** Demographic and clinical characteristics of HIV-infected participants.

	NC (*n* = 8)	ANI (*n* = 17)	MCMD (*n* = 25)	HAD (*n* = 11)	*p*-value (Intra-CI)	All CI (*n* = 53)	*p*-value (NC *vs* CI)
Age, years	38.3 [35.1, 57.1]	49.0 [37.4, 51.8]	50.3 [39.6, 53.8]	51.6 [38.5, 55.1]	0.45	50.0 [39.2, 53.8]	0.73
Male [*n* (%)]	6 (75.0%)	14 (82.4%)	25 (100%)	11 (100%)	**0.03**	50 (94.3%)	0.24
Education (years)	12.0 [12.0, 14.5]	14.0 [12.0, 16.0]	14.0 [12.0, 16.0]	14.0 [12.0, 14.0]	0.69	14.0 [12.0, 16.0]	0.23
NPZ global score	0.4 [0.2, 0.5]	−0.3 [−0.7, −0.0]	−0.5 [−0.9, 0.2]	−1.0 [−1.1, −0.5]	0.08	−0.50 [−0.9, 0.1]	**<0.001**
CD4+ count (cells/μL)	494 [213, 645]	429 [335, 739]	533 [333, 649]	175 [125, 85]	**<0.001**	429 [285, 570]	0.94
CD4+ nadir (cells/μL)	265 [107, 339]	351 [120, 730]	180 [74, 313]	55 [16, 85]	**0.002**	180 [71, 351]	0.63
HIV duration, years	9.0 [5.1, 11.6]	7.5 [1.1, 15.9]	12.1 [7.7, 17.2]	10.8 [7.9, 14.3]	0.243	10.8 [5.7, 16.4]	0.42
Undetectable HIV RNA, *n* (%)	5 (62.5%)	10 (58.8%)	14 (56.0%)	8 (72.7%)	0.632	32 (60.4%)	0.40

Results are reported as median with interquartile ranges. Intra-CI comparisons (ANI vs MCMD vs HAD) were examined by KruskalWallis and chi-squared tests for continuous and categorical data, respectively. Comparisons between NC and CI groups were performed by Mann–Whitney U and chi-squared tests for continuous and categorical data. NC, normal cognition; ANI, asymptomatic neurocognitive impairment; MCMD, minor cognitive motor disorder; HAD, HIV-associated dementia. NPZ global score, neuropsychological z score. Bold values indicate the statistically significant.

### EVs expressing putative bone and kidney markers are associated with cognitive impairment in PWH on suppressive ART

**Figure 2:**
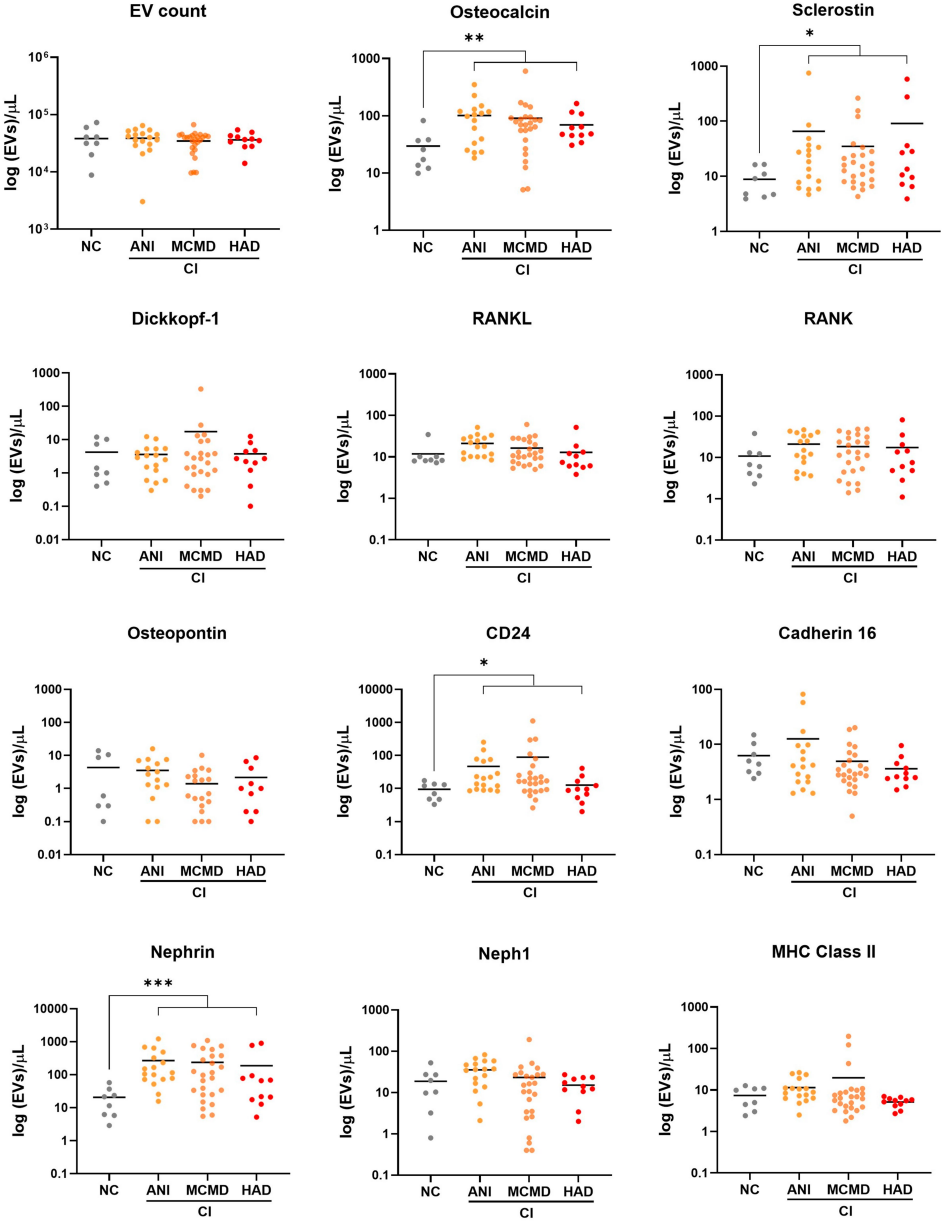
Circulating EVs bearing bone and kidney cell markers associate with cognitive impairment in PWH. Scatter plots of EV concentration (log 10-transformed). Levels of EVs expressing bone-associated markers and kidney markers were significantly higher in PWH with cognitively impaired (CI) compared to PWH with normal cognition (NC). *p* values were determined by using two-tailed Mann–Whitney test. *p < 0.05, **p < 0.01, ***p < 0.001. ANI, asymptomatic neurocognitive impairment; MCMD, minor cognitive motor disorder; HAD, HIV-associated dementia.

**Figure 3:**
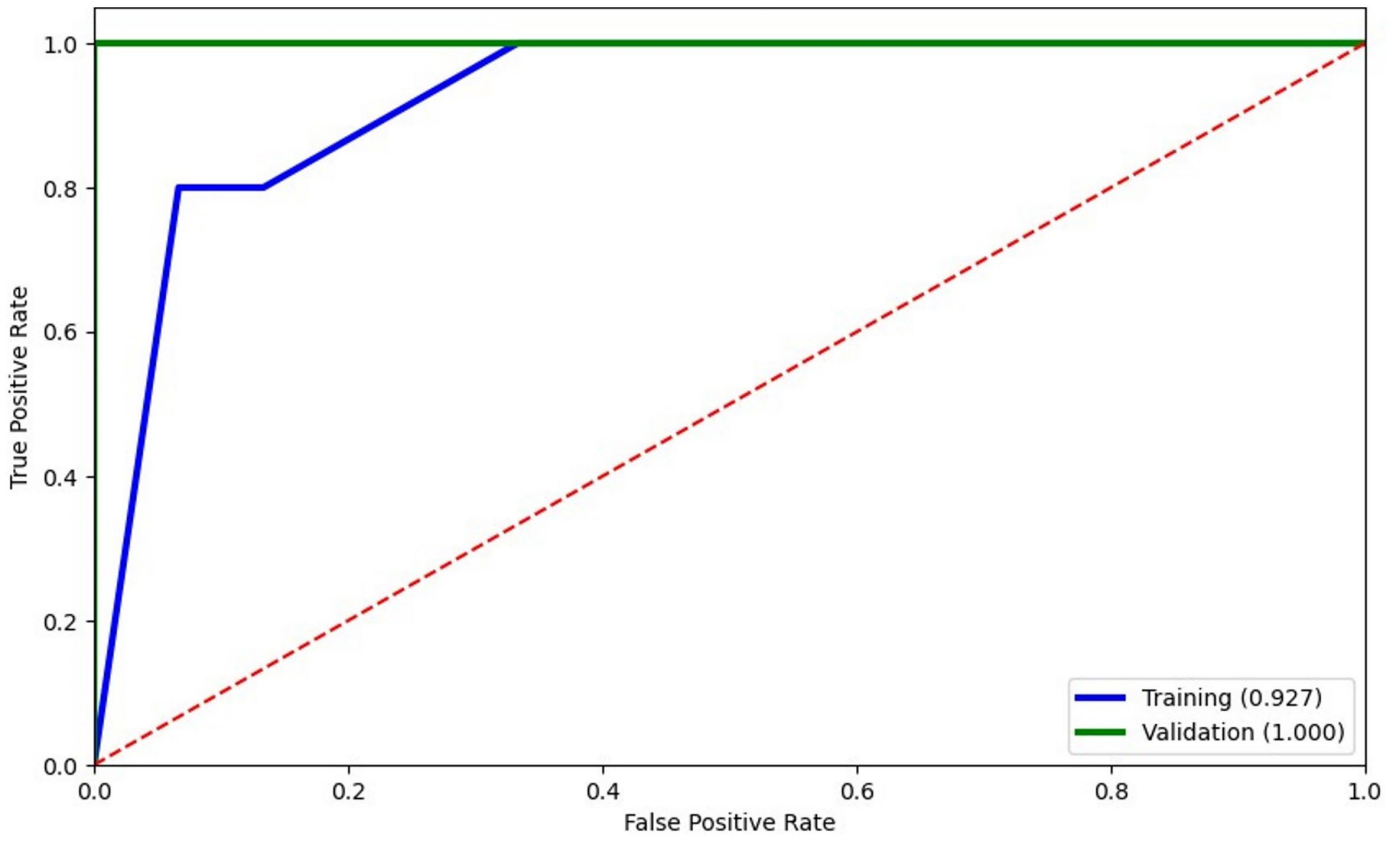
Logistic regression modeling of EVs expressing bone and kidney markers to predict cognitive status. Training of the model (blue) was well trained with an accuracy score of 0.927 and scored 100% accuracy on validation. The XGBoost model selected only 3 features: osteocalcin+EVs, nephrin+EVs, and CD24 + EVs.

Considering that potential markers of cellular activation and neuroinflammation were significantly abundant on EVs from HIV+ individuals with CI (13), we next determined whether levels of EVs from bone-and kidney-related cells associate with cognitive dysfunction. The abundance markers of plasma-EVs from PWH with and without CI are shown in Table 2. There were no significant differences in the total EV concentration between the NC and CI groups. Plasma levels of EVs expressing dickkopf-1, RANKL, RANK, osteopontin, cadherin-16, Neph1, and MHC Class II did not statistically differ between the groups. However, we observed significant elevations in levels of EVs expressing two of the bone-associated markers, osteocalcin and sclerostin, as well as EVs expressing the kidney-associated markers, nephrin and CD24, in HIV-infected individuals with cognitive impairment compared to those with normal cognition (Figure 2). When adjusted for multiple comparisons, osteocalcin, sclerostin, and nephrin remained significant (p_adj_ ≤ 0.077). Finally, to assess the power of osteocalcin-, sclerostin-, and nephrin-expressing EVs by cognitive status, an XGBoost model was trained and validated on only participants with viral load <1,000 copies/mL. The resulting model had an accuracy score of 0.927, suggesting the model was well trained. The validation subset produced an accuracy of 100% (Figure 3). These results suggest that osteocalcin+, nephrin+, and CD24+ EVs may serve as novel biomarkers of HIV-related cognitive disorders.

**Table 2:** Abundance of plasma-derived EVs from PWH by cognitive status.

	NC (*n* = 8)	ANI (*n* = 17)	MCMD (*n* = 25)	HAD (*n* = 11)	*p*-value (Intra-CI)	All CI (*n* = 53)	*p*-value (NC *vs* CI)	P_adj_
Total EV count/μL	10.5 [10.2, 10.7]	10.6 [1.9, 2.4]	10.6 [10.2, 10.7]	10.5 [10.3, 10.6]	0.64	10.5 [10.3, 10.7]	0.93	0.93
MHC Class 2 + EVs/μL	2.0 [1.4, 2.4]	2.2 [1.9, 2.4]	1.9 [1.4, 2.2]	1.7 [1.5, 1.8]	0.40	1.9 [1.6, 2.3]	0.92	0.93
Dickkopf-1 + EVs/μL	0.2 [−0.3, 2.1]	1.1 [0.2, 1.7]	0.7 [−0.1, 1.4]	1.0 [0.5, 1.5]	0.55	1.0 [−0.1, 1.5]	0.80	0.93
Osteocalcin + EVs/μL	3.1 [2.6, 3.6]	4.6 [3.5, 4.8]	4.3 [3.6, 4.5]	3.9 [3.8, 4.4]	0.70	4.2 [3.6, 4.7]	**0.003**	**0.02**
Osteopontin + EVs/μL	−0.9 [−1.6, 2.2]	0.6 [−0.8, 1.7]	−0.7 [−2.4, 0.6]	−0.0 [−1.8, 0.9]	0.11	−0.0 [−2.4, 1.2]	0.68	0.90
Sclerostin + EVs/μL	1.9 [1.5, 2.5]	2.9 [2.1, 3.5]	2.7 [2.1, 3.2]	2.6 [2.1, 3.5]	0.50	2.7 [2.1, 3.4]	**0.02**	**0.08**
RANKL + EVs/μL	2.1 [2.1, 2.3]	2.9 [2.3, 3.3]	2.4 [2.0, 3.1]	2.0 [1.8, 2.5]	0.22	2.5 [2.1, 3.2]	0.23	0.39
RANK + EVs/μL	1.9 [1.4, 2.5]	2.7 [2.0, 3.5]	2.6 [1.5, 3.4]	2.0 [1.6, 2.8]	0.83	2.6 [1.6, 3.4]	0.20	0.39
CD24 + EVs/μL	2.2 [1.6, 2.6]	3.0 [2.3, 4.2]	2.8 [2.2, 3.4]	2.3 [1.8, 2.7]	0.40	2.7 [2.2, 3.4]	**0.04**	0.12
Cadherin16 + EVs/μL	1.5 [1.2, 2.0]	1.4 [0.8, 2.3]	1.1 [0.8, 1.6]	1.0 [0.9, 1.4]	0.12	1.1 [0.8, 1.8]	0.17	0.39
Nephrin + EVs/μL	2.8 [1.8, 3.3]	4.7 [4.3, 5.8]	4.6 [3.2, 5.9]	4.2 [3.0, 4.4]	0.81	4.5 [3.2, 5.8]	**<0.001**	**0.01**
Neph1 + EVs/μL	2.7 [2.0, 3.3]	3.6 [2.8, 3.9]	2.7 [1.2, 3.3]	2.7 [2.4, 3.1]	0.19	2.9 [2.2, 3.5]	0.61	0.90

Results are reported as median with interquartile ranges. EV measures are the log of EVs/μL. Intra-CI comparisons (ANI vs MCMD vs HAD) were examined by Kruskal-Wallis and chi-squared tests. Comparisons between NC and CI groups were performed by Mann–Whitney U. benjamini-Hochberg adjustments for multiple comparisons between NC and CI groups are represented by adjusted *p*-values (P_adj_). NC, normal cognition; ANI, asymptomatic neurocognitive impairment; MCMD, minor cognitive motor disorder; HAD, HIV-associated dementia. Bold values indicate the statistically significant.

**Figure 4:**
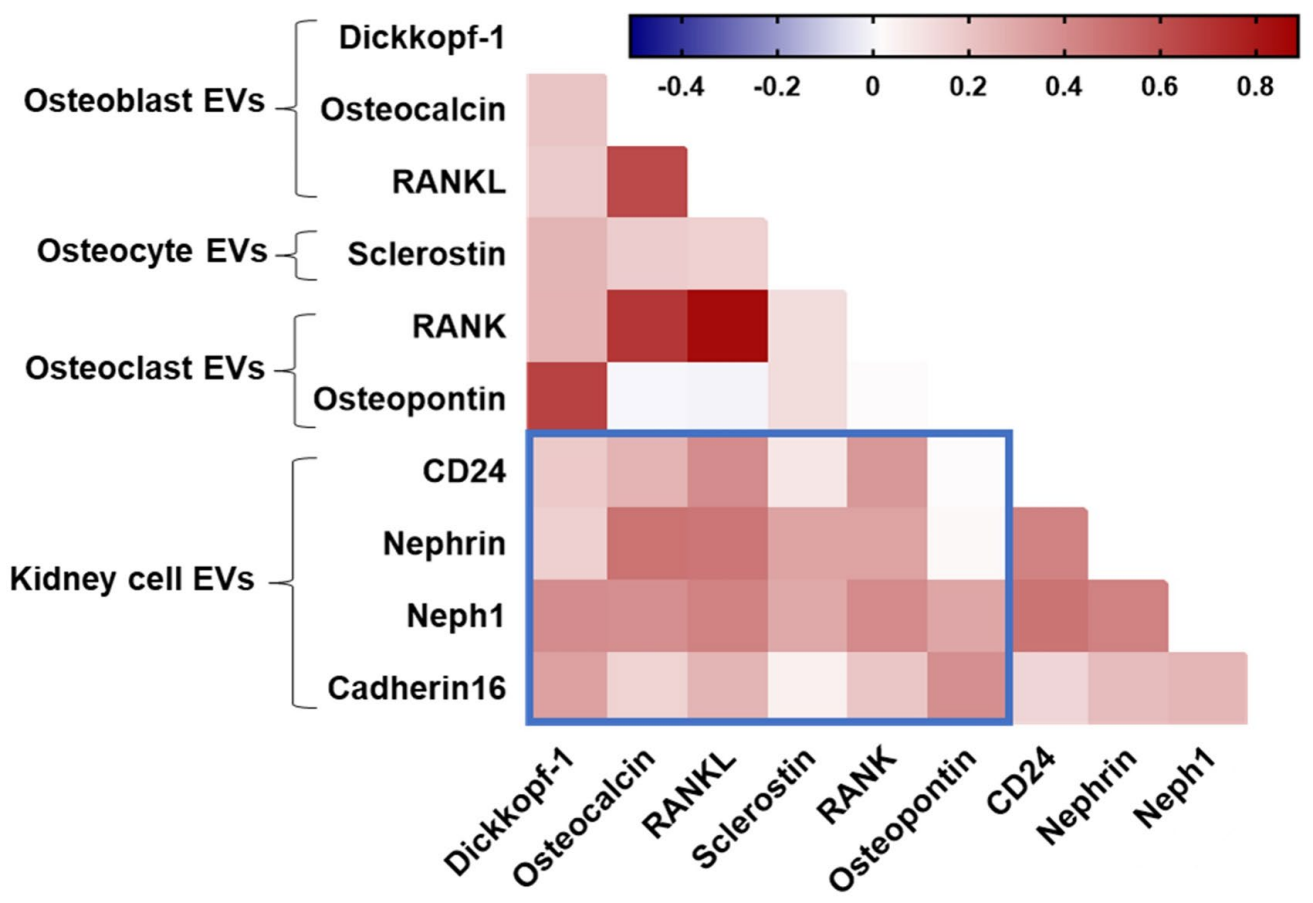
Spearman correlation between EV subtypes. Blue boxes denote correlations between bone and kidney EVs. Positive correlations are displayed in red shades.

Finally, we explored the relationship between bone-derived EVs with kidney-derived EVs (Figure 4). Using Spearman’s rank correlation, a positive relationship was observed between levels of EVs expressing the bone markers osteocalcin, RANKL, and RANK with kidney-derived EVs (neph1, nephrin, and CD24). We also observed a correlation between levels of osteoblast-associated markers (dickkopf-1 and osteopontin) derived from EVs with Neph1 + EVs and cadherin-16 + EVs. There was a relationship between levels of EVs expressing the osteocyte-derived sclerostin with glomerular epithelial markers (Neph1 + EVs and nephrin+EVs). These results remained significant after correcting for multiple testing with an adjusted *p*-value (p_adj_) < 0.1. Taken together, these associations might suggest that bone and kidney cell-derived EVs may be coordinately regulated in the pathophysiology of HIV-related cognitive impairment.

## Discussion

To the best of our knowledge, this study provides the first evidence that levels of EVs enriched with the bone markers osteocalcin and sclerostin and the kidney marker nephrin are associated with cognitive impairment in PWH on suppressive ART, suggesting that higher abundance of EVs expressing these proteins may serve as novel candidate biomarkers for disease-spanning neurocognitive dysfunction and might lay the foundations of signaling mechanisms between the nervous system and other organs. Consistent with our observations, some studies have demonstrated that bone-secreted factors could influence cognitive function. Ross et al. reported that bone-derived osteocalcin was associated with worse working memory in women living with HIV, and plasma sclerostin levels trend toward an association with cognitive decline (8). Liu et al. reported that brain-derived EVs from Alzheimer’s disease mice can across the blood–brain barrier to reach the bone tissue and promote a bone-fat imbalance. Further, level of EV miR-483-5p was associated with bone loss in Alzheimer’s disease patients, which might mediate neuronal tissue controls of bone remodeling during disease (16). Another study performed by Xia et al. (23) showed that EVs and their miRNA cargo derived from patients and rats with injured neurons can target osteoprogenitors to promote osteogenesis, suggesting a neuroimmune crosstalk in the bone-brain axis during bone homeostasis. Furthermore, consistent with this notion, a recent study reported that EV miR-34a had the same trend of expression in the brain and the kidney of mice deficient in protein L-isoaspartyl methyltransferase, suggesting that EVs may mediate the exchange of signals in the kidney-brain axis (24). Although our study did not investigate the EV cargo, it is important to mention that circulating EVs, particularly their miRNA content, may shed light on this aspect of interorgan crosstalk and indicate that certain EV cargo may play a role in the neuropathological conditions of tissues, encompassing both in the brain and other organs. Taken together, our recent findings and these observations suggest that EVs from bone-and kidney-related cells may take part in the pathophysiological process of HIV-related cognitive impairment and mediate the functional crosstalk in near-and long-distant organs.

Considering the machine learning model for identifying cognitive phenotypes in PWH, osteocalcin+EVs, nephrin+EVs, and CD24 + EVs accurately predicted cognitive dysfunction with 93% certainty within PWH with viral load <1,000 copies/mL. Osteocalcin has emerged as an osteoblast-specific protein with potential endocrine properties that can across the blood–brain barrier and affect neuronal function (25). Collectively, several studies using mouse models observed that osteocalcin can enhance neurogenesis and cognitive performance (25–27). Another interesting point is that increased levels of osteocalcin were observed in individuals with neurodegenerative diseases, including Alzheimer’s disease and Parkinson’s disease (28–31). Along with the bone-derived osteocalcin, the osteocyte-derived sclerostin may regulate the Wnt/β-catenin signaling pathway, which is essential for regulating bone metabolism and cognitive function and has been associated with Alzheimer’s disease progression (14, 32, 33). Furthermore, the association between elevated levels of plasma sclerostin and high brain amyloid-β deposition in older individuals at high risk of Alzheimer’s disease may be proposed as a crosstalk between bone and brain (34). In addition, some studies observed that nephrin, a protein expressed in glomerular podocytes, could serve as a predictive marker for renal damage and neuronal dysfunction in mouse models of diabetic kidney disease (35–39).

It is important to mention that some studies have pointed out that EVs could serve as potential therapeutic tools for several conditions, such as neurological disorders, and cardiac and bone diseases, as they can target multiple pathways and carry anti-inflammatory, antioxidant, pro-angiogenic, and anti-apoptotic properties. However, a recent study identified 60 unique clinical trials on clinicaltrials.gov underscores the diverse range of conditions being targeted with EV-based therapies, including moderate-to-severe COVID-19 infection, chronic post-COVID 19 syndrome, Alzheimer’s disease, acute myocardial infarction, Type I diabetes, cancer, and osteoarthritis (40). These comprehensive approaches observed in clinical trials may reflect the potential of EVs in addressing a wide array of health conditions.

While the strengths of this study include the utilization of the clinical and demographic data from the longitudinal HAHC cohort (17), our study has limitations; first, this study was limited by modest sample size and the lack of investigating soluble bone remodeling proteins as well as bone mineral density, which might have detected the association between bone health and cognitive function in PWH. Another limitation was the lack of information on other variables such as glomerular filtration rate, creatinine, and albumin to explore the relationship between renal function and cognitive status. Further investigations are also required to validate our findings through a larger cohort as well as measurement of EV subtypes in similar aged HIV uninfected individuals. However, whether the development of milder forms of HIV-associated neurocognitive disorders is caused (at least in part) by bone-derived EVs in the brain or whether EV signaling factors, including osteocalcin and sclerostin, could contribute to restoring cognitive functions remains to be investigated. Furthermore, elucidating the mechanisms governing their crosstalk between the brain and other organ systems could provide potential targets for therapy not just for HAND but for other degenerative diseases in the general population.

## Data availability statement

The original contributions presented in the study are included in the article/Supplementary material, further inquiries can be directed to the corresponding author.

## Ethics statement

The studies involving humans were approved by the Institutional Review Board of the University of Hawaii Committee on Human Subjects and conducted in accordance with the local legislation and institutional requirements. The participants provided their written informed consent to participate in this study.

## Author contributions

EM: Conceptualization, Formal analysis, Investigation, Methodology, Project administration, Writing – original draft. SB: Formal analysis, Writing – review & editing. CS: Resources, Writing – review & editing. LN: Conceptualization, Formal analysis, Resources, Investigation, Methodology, Supervision, Writing – review & editing. PN: Conceptualization, Formal analysis, Resources, Investigation, Methodology, Supervision, Writing – review & editing.
